# Enhanced precision in cell culture analytics: leveraging artificial intelligence for unbiased and non-destructive assessment of cell growth and viability

**DOI:** 10.1038/s41420-026-03116-9

**Published:** 2026-04-13

**Authors:** Cheung Pang Wong, Nasrin Khazamipour, Soroush Aalibagi, Sukrit Kapoor, Louise Ramos, Joya Maria Saade, Casper Dolleris, Janny Marie L. Peterslund, Daria Golnarian, Negin Farivar, Mads Daugaard, Nader Al Nakouzi

**Affiliations:** 1 Vancouver Prostate Centre, Vancouver, BC Canada; 2 SnapCyte Solutions Inc., Vancouver, BC Canada; 3 Department of Urologic Sciences, University of British Columbia, Vancouver, BC Canada

**Keywords:** Assay systems, Cell biology

## Abstract

Precise assessment of cell growth, count, and viability is a prerequisite for biological and medical research. Traditional cell analytics involve manual processes, such as cell counting or reagent-based approaches that are user-dependent and prone to bias. Semi-automated systems for counting cells, tracking cell growth, and determining viability have been introduced over the past decades. However, these methods are often time-consuming, require labeling steps, and involve costly instrumentation and consumables. Changes in cell growth and/or viability create biological patterns that can be interpreted by artificial intelligence (AI). Here, we report the development and validation of SnapCyte™, an AI application that performs accurate, unbiased, label- and reagent-free cell analyses from basic cell culture images. Using cell lines with diverse morphologies in various culture conditions, we generated a comprehensive and fully annotated image database that was used for AI education. Convolutional neural networks were employed for cell localization and iterative training loops until a stable performance of >95% accuracy was obtained for all readouts. The fully trained AI demonstrated high Precision and Recall and performed with greater accuracy and less variation as compared to standard methods. As the SnapCyte analyses are performed on cell images only, data acquisition is non-invasive to the experimental setup, enabling real-time use of cells in downstream assays. In summary, SnapCyte is a fast and accurate cell analytics platform, resistant to user variations and independent of reagents or specific equipment, with improved performance over current cell analytics methodologies.

## Introduction

Routine quantification of confluency, cell counts, and cell viability is a critical step in life science research and biopharmaceutical industry workflows [1–3]. Accurate and consistent measurements of these parameters are essential for data reproducibility and experimental success. Confluency refers to the percentage of a surface area covered by a layer of cells in a culture vessel [2]. Regular determination of confluency is commonly used as a readout for cell growth and fitness in research fields such as cancer biology, stem cell research, and regenerative medicine [4, 5]. In any experimental setup, maintaining optimal cell density is crucial, as high confluency can affect growth dynamics, delay cell passaging and harvesting, and impact downstream experiments. Overcrowding can limit nutrient access, reduce cell viability, and cause cell detachment from the culture plate surface [6]. Conversely, low confluency can result in insufficient cell-to-cell contact, impairing cell signaling and growth [6, 7]. Growth dynamics and cell concentrations depend on inoculum cell count [7, 8]. Cell counting is used for determining seeding density but also for tracking cell growth rates, particularly in non-adherent cell cultures, providing information on cellular fitness and/or responses to experimental treatments. Obtaining precise cell numbers is required for seeding cells in culture plates to be used in subsequent experimental setups, normalizing results, and optimizing experimental conditions for procedures such as cell transfections [9, 10]. In clinical settings, precise cell counts are crucial for determining healthy ranges of cell populations, assessing toxicity, or evaluating immune reactions and other clinical parameters. Accurate cell counts are therefore vital for both life science research and clinical outputs [11, 12].

Tracking cell growth is instrumental for evaluating the fitness of cell cultures, and the proliferation of live cells serves as an important indicator of culture conditions. Changes in growth rates can provide information on cellular responses to stimuli or drug treatments. Given its importance, there are various methods for measuring cell growth. They can roughly be categorized into manual, colorimetric, and image-based methods. Examples of these methods include chemical dyes (e.g., MTT (3-(4,5-dimethylthiazol-2-yl)-2,5-diphenyltetrazolium bromide) assay), manual enumeration [13], automated cell counters [14], manual image analyses (e.g., ImageJ) [15], and automated image processing methods (e.g., Incucyte®) [16]. While these techniques are valuable, the readouts are affected by human bias and variability in experimental conditions. Conventional determination of cell counts and cytometric parameters, most prominently, alive and intact dead cell ratios, is performed using a hemocytometer (e.g., Bürker-Türk counting chamber) [17, 18]. The major advantage of this conventional approach is the direct observation of the cell culture by the operator, which enables rapid detection of problems such as contamination or aggregation of cells. However, this method is prone to human error that can occur during many stages of the cell enumeration process, including mixing, handling, and dilution of the cells. Biggs et al. performed a study to quantify the standard error among trained technicians who counted red blood cells from one sample of blood using hemocytometers [19]. They reported that the standard errors between the results of individual experiments conducted by two technicians and five technicians were 3.6% and 7.6%, respectively, suggesting that the magnitude of errors increases with the number of individuals in an experiment. Different researchers might interpret mammalian cell boundaries in a hemocytometer differently, impacting results. A more recent study conducted by Manzini et al. showed that the variation can reach nearly 20% among different operators who are highly experienced in cell counting [20]. Another report documented the effects of dilution factors on total cell concentration measurements from manual methods. The coefficients of variation surged from 0.072 to 0.2 between 0.3 and 0.5 dilution fractions in an experiment, indicating the degree of human error in diluting samples during the cell counting process [21]. Additionally, the reproducibility of manual cell counts can be low, especially if high cell density cultures are used [22]. Beyond the issue of human error, manual counting of cells is also a time-consuming task, especially when having to perform multiple counts on the same culture for increased accuracy. This can lead to process fatigue and cause errors due to the subjective interpretation of borderline cases.

Colorimetric assays, such as MTT (3-(4,5-dimethylthiazol-2-yl)-2,5-diphenyltetrazolium bromide) and crystal violet, are widely used for assessing cell proliferation due to their extensive validation in numerous publications. However, these methods have notable limitations. The MTT assay measures metabolic activity by relying on the reduction of MTT by mitochondrial dehydrogenases to form an insoluble formazan product. This method is influenced by factors such as mitochondrial activity, which may not directly correlate with cell number or viability, leading to inaccuracies. Also, compromised metabolic activity in viable cells can yield misleading results [13]. Crystal violet staining, which involves binding of dye to DNA in viable cells, is semi-quantitative and influenced by cell size and DNA content variations. It does not distinguish between live and dead cells, potentially overestimating proliferation [23]. Both MTT and crystal violet assays are destructive, requiring cell lysis that prevents further analysis of the same cell population. Additionally, the definitive endpoint of the assay precludes real-time monitoring of growth and viability, calling for multiple parallel setups that increase the risk of error. The use of plate readers adds to the complexity and cost. Other reagent-based assays, such as WST-1 and CyQuant, offer some advantages but not without notable limitations. The WST-1 assay measures the conversion of a tetrazolium salt to a water-soluble formazan product, which is a more stable reaction compared to MTT. However, the assay still depends on cellular metabolic activity, which can vary independently of cell number [24]. CyQuant assays use a fluorescent dye that binds nucleic acids, providing a more direct and sensitive measurement of cell number. However, CyQuant requires cell lysis, limiting subsequent live-cell analyses [25]. Collectively, reagent-based methods for assessing cytostasis or cytotoxicity are often static or indirect, evaluating treatment effects at single time points or on associated cellular processes, such as membrane integrity. These methods cannot capture dynamic changes over time, and they rely on endpoint measurements that do not reflect temporal variations in cell growth or viability. These limitations highlight the need for more accurate, dynamic, and non-destructive methods for assessing cell growth and viability in cell cultures.

Image-based methods measuring cell confluency (instrument-linked methods such as IncuCyte®) have gained popularity in recent years. While offering the convenience of automation and real-time tracking, these instruments are costly, requiring consumables and maintenance, and have limited user capabilities, making them inaccessible for many laboratories. Other image-based methodologies have also been developed using image processing, or more recently, machine learning (ML) to overcome user variability and reduce processing time, but they come with their own limitations. For example, the method developed by Soleimani et al. involves a multi-step process with image normalization, contrast enhancement, denoising, binary image conversion, modification, and finally measurement [26]. This method’s empirical modification step, akin to morphological opening [27], may lead to incorrect cell and noise classification, as manual adjustments in image processing can introduce bias and misclassification. Other methods struggle with extreme density scenarios. Wang et al. reported low accuracy (F-score <80%) for low confluency images (<40%) and the method relied on artificial rules, such as thresholds, which hindered algorithm generalizability [28]. Furthermore, the deep learning-based approach presented by Ayanzadeh et al. [29] shows great potential but focuses solely on low confluency cases, neglecting high confluency scenarios, thereby highlighting the need for improved methods. Overall, while current image-based methods offer advancements over reagent-based techniques, they require further development to address limitations and improve accuracy and accessibility for a wider range of laboratories. Transitioning from these traditional methods, deep learning-based computer vision emerges as a superior approach, setting new benchmarks in image-based cell analytics. Although there have been attempts to integrate various ML technologies into cell analysis, these methods require further enhancement to accommodate precise detection of clumped or overlapping cells, and adaptability to different cell morphologies and imaging conditions [30–32]. For instance, the methodology developed by Jiang et al. employs a random forest and density map framework utilizing handcrafted features that limit its performance due to a limited receptive field [30]. Similarly, the approach by Schmidt et al. uses a density-regression deep convolutional neural network, combining a U-Net-like density predictor with a VGG-like regression model [31]. Although innovative, this approach struggles with detecting clumped and overlapping cells. Cellpose offers a robust segmentation algorithm that performs well across various cell types with minimal adjustments [32]. However, this method falters under varying imaging conditions and morphological changes in our experiments, affecting its accuracy and limiting its general applicability to different data types. These limitations underscore the need for more robust and objective methods for cell growth quantification. Here, we describe the development and performance of a novel AI platform technology for advanced cell analytics that can be easily integrated with most laboratory workflows.

## Results

### Deep learning optimization for cell confluency detection

**Fig. 1:**
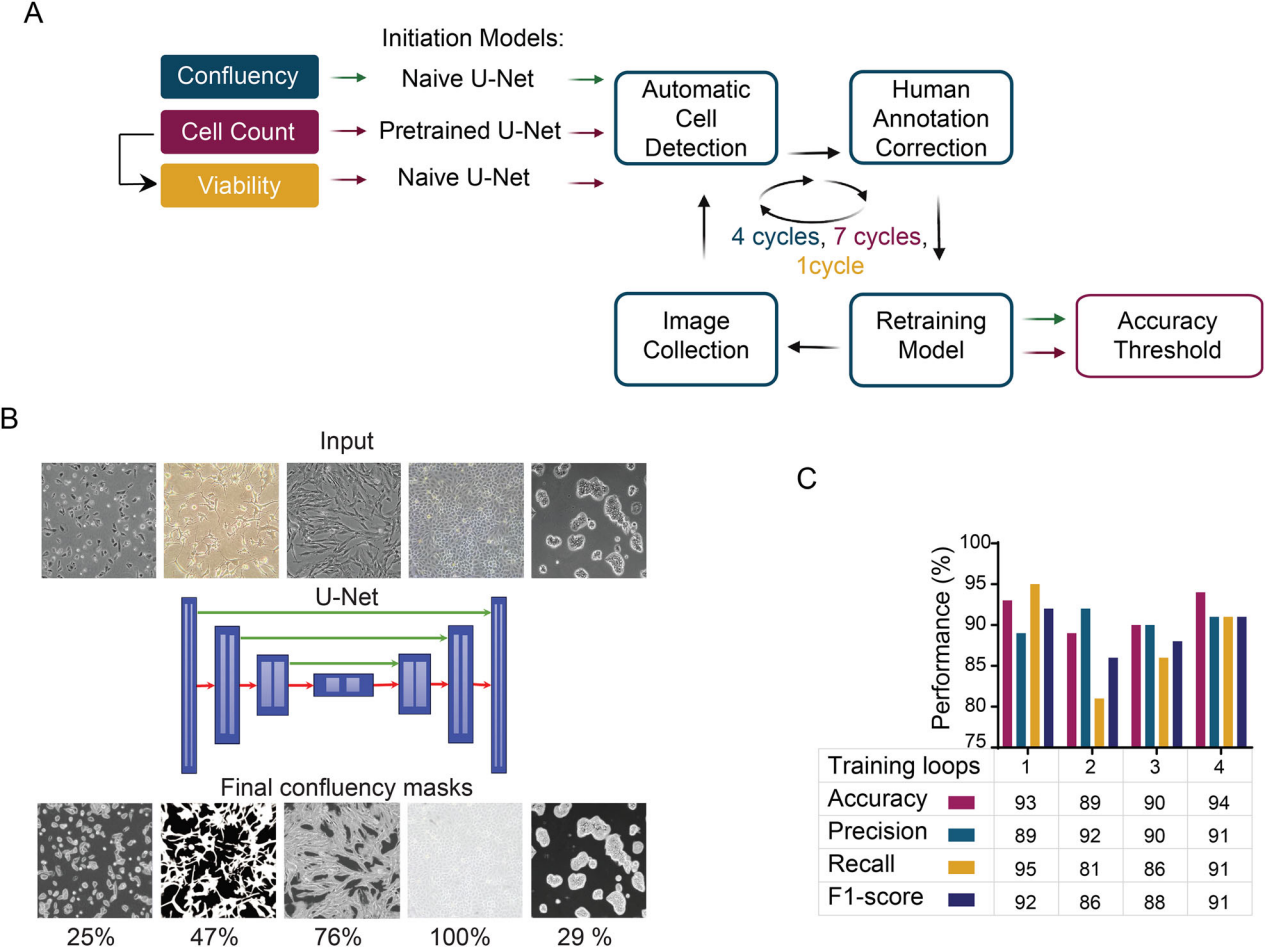
Deep learning optimization for cell confluency detection. **A** A visual overview of SnapCyteTM ML processes and development strategy. An iterative human-in-the-loop approach is used for fine-tuning the confluency, cell count, and viability models, starting with a naive U-Net model for confluency and viability and a pretrained Cellpose model architecture and Cyto weights for cell count. Multiple training cycles were needed until the desired thresholds for Accuracy, Precision, Recall, and F1 score were achieved. **B** The cell confluency machine learning architecture is a standard backbone U-Net with downsampling and upsampling paths and skip connections. Representative input images and their corresponding SnapCyteTM-generated masks and confluency values (%) are shown. **C** Machine learning performance parameters for the cell confluency model during the training iterations until a 90% threshold was reached for all parameters.

Cell growth patterns are reflected in the numbers and densities of cells in a defined area or volume. These patterns can be captured in microscope images that are also particularly well-suited for AI assessment. To build an AI capacity able to assess growth patterns of cells, we designed a simple workflow that utilized an iterative human-in-the-loop approach for fine-tuning the confluency. Starting with a naive U-Net model, multiple training cycles using human-annotated and corrected sets were needed to ultimately reach output performances of >90% accuracy (Fig. 1A). We first determined an AI model that could adequately report on cell density or confluency as a readout for cell growth. We employed a convolution-based encoder-decoder architecture based on U-Net for training of the AI (Fig. 1B) [33]. We utilized Accuracy, Precision, Recall, and F1-score parameters to assess the pixel-level segmentation performance of our model quantitatively. The Accuracy parameter provides a comprehensive measure of segmentation performance on both foreground and background [34], while the F1 score focuses on the accuracy of target segmentation by the network [35]. Following four training iteration cycles, the model achieved a desirable threshold of Accuracy (0.94) and F1 (0.91) (Fig. 1C).

### Robustness of confluency detection across heterogeneous imaging conditions and cell types

**Fig. 2:**
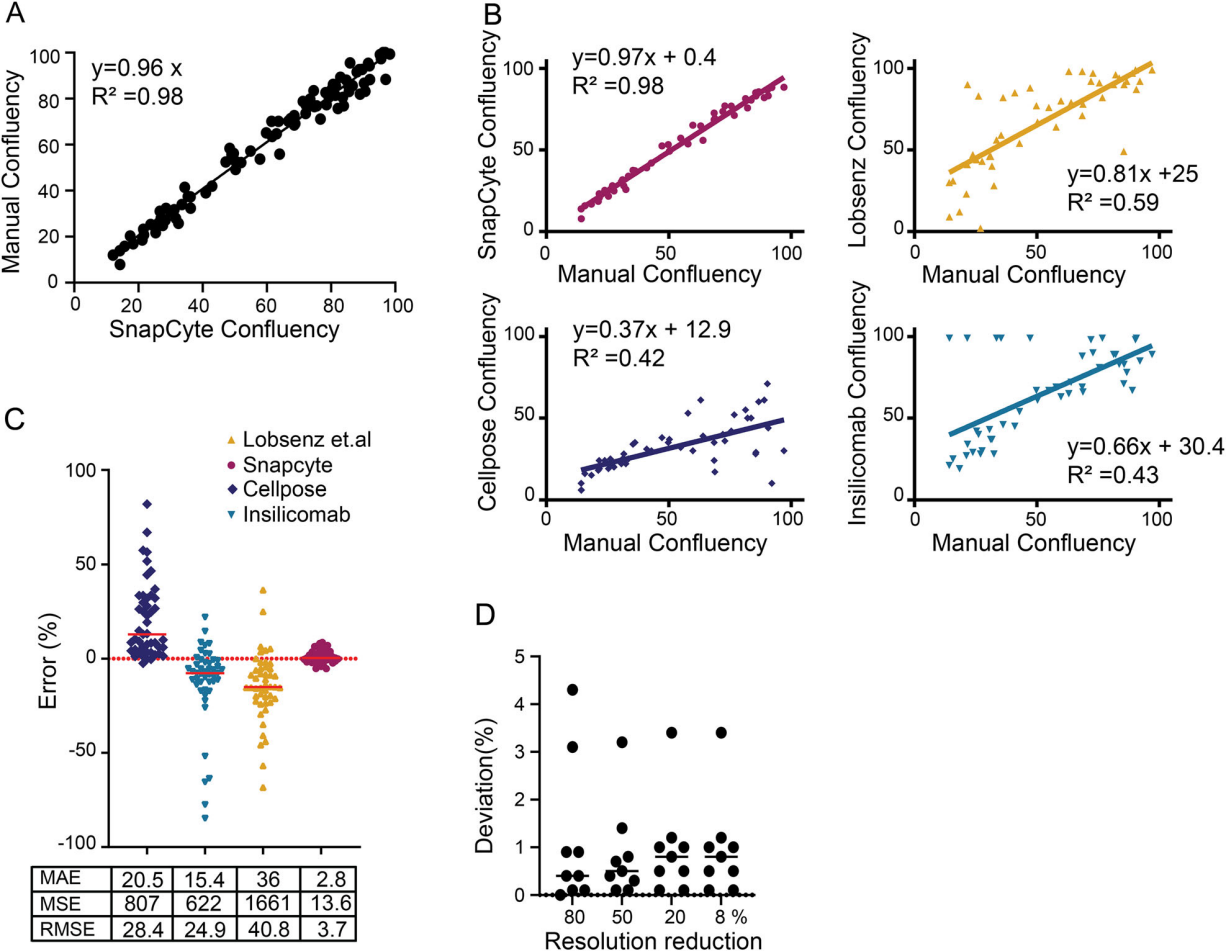
Robustness of confluency detection across heterogeneous imaging conditions and cell types. **A** Correlation between SnapCyteTM mask and expert manual annotations of 86 images, 50% of which are 23 commonly used ATCC cell lines evaluated at low-to-high confluency levels, and the remaining 50% of them are images of 42 cultures reported in publications. **B** Correlation between confluency values generated by different models (SnapCyte, Lobsenz, Cellpose, and Insilicomab) and manually determined confluency using a dataset of 42 images from 23 commonly used ATCC cell lines. Each plot shows the linear regression equation and the coefficient of determination (R-squared). A slope close to 1 and a high R-squared value indicate strong agreement between the model-generated and manual confluency values. SnapCyte achieves the highest correlation (*R*^2^ = 0.98) and a slope close to 1, reflecting near-perfect alignment with manual measurements. Other models exhibit varying levels of correlation and accuracy. **C** Error analysis of confluency predictions by Lobsenz, SnapCyte, Cellpose, and Insilicomab models. Scatter plots display the distribution of percentage errors. The table summarizes the Mean Absolute Error (MAE), Mean Squared Error (MSE), and Root Mean Squared Error (RMSE) for each model, highlighting SnapCyte’s superior accuracy and lowest error metrics across all categories. **D** Re-sampling of 10 different cell line images (i.e., MCF7, MG63, LNCaP, VCaP, IGR-CaP1, NCIH660) to 80%, 50%, 20%, and 8% of their original resolution and analyzed using SnapCyteTM. The deviation was less than 1%, indicating extreme robustness towards variations in image quality.

Since the SnapCyte ML model is designed to be user-independent, with no need for users to set parameters, its universality is crucial. This means the model must perform effectively with images acquired in diverse settings and across a wide range of cell types. In the first step, we compiled and annotated a test set of 86 images, covering a broad spectrum of image qualities and sizes. This test set consisted of 43 published images of ATCC’s top 23 cell lines, available at both low and high confluency, and 43 images from 42 cell lines, as shown in 18 publications [36–52]. SnapCyte analyzed all images, generating masks that were compared to HQP annotation masks (Fig. 2A and Supp Tables 1, 2). SnapCyte achieved a strong correlation with manual measurements (*R*^2^ = 0.98) and a slope of 0.96, reflecting near-perfect alignment. We further tested SnapCyte against existing ML models using the ATCC image dataset (*n* = 41), including a modified Cellpose model [32], the approach described in Lobsenz et al. [53], and a widely accessible community-contributed method “Insilicomab confluency tool” (https://github.com/insilicomab/cell_confluency). Our analyses demonstrated a strong correlation between manually assessed confluency values and those generated by SnapCyte at both high and low cell density conditions, with an *R*² of 0.98. In contrast, the Cellpose, Lobsenz, and Insilicomab confluency tool models showed weaker correlations, with *R*² values of 0.59, 0.42, and 0.43, respectively (Fig. 2B). Raw images together with their corresponding annotated segmentation masks and quantitative outputs are provided in Supplementary Tables 1 and 2, allowing independent visual inspection and verification of segmentation accuracy We also assessed the error metrics where the Mean Absolute Error (MAE) of SnapCyte was significantly lower at 2.8, indicating exceptional accuracy with minimal deviation from actual values. Conversely, the other models displayed considerably higher MAEs of 15.4, 20.5, and 36, suggesting less precise predictive capabilities and significant average errors. The Root Mean Squared Error (RMSE) for SnapCyte was also low at 3.7, highlighting the model’s accuracy and error consistency. The other models, such as Insilicomab confluency tool, Lobsenz, and Cellpose, recorded RMSEs of 24.9, 28.4, and 40.8, respectively, indicating moderate to significant errors that could compromise model reliability and effectiveness in practical applications (Fig. 2C).

Furthermore, we explored the model’s robustness concerning image resolution by analyzing 10 images of diverse cell lines (e.g., MCF7, MG63, LNCaP, VCaP, IGRCaP-1, NCIH660) at 80%, 50%, 20%, and 8% of their original resolutions. The confluency value deviations between these resolutions were less than 2% (Fig. 2D), showcasing the model’s robustness against variations in image quality. Overall, our data demonstrate that SnapCyte maintains high robustness, universality, and independence from experimental settings such as microscope lighting or image resolution.

### Optimizing image sampling for efficient and accurate confluency estimation in cell culture vessels at various magnifications

**Fig. 3:**
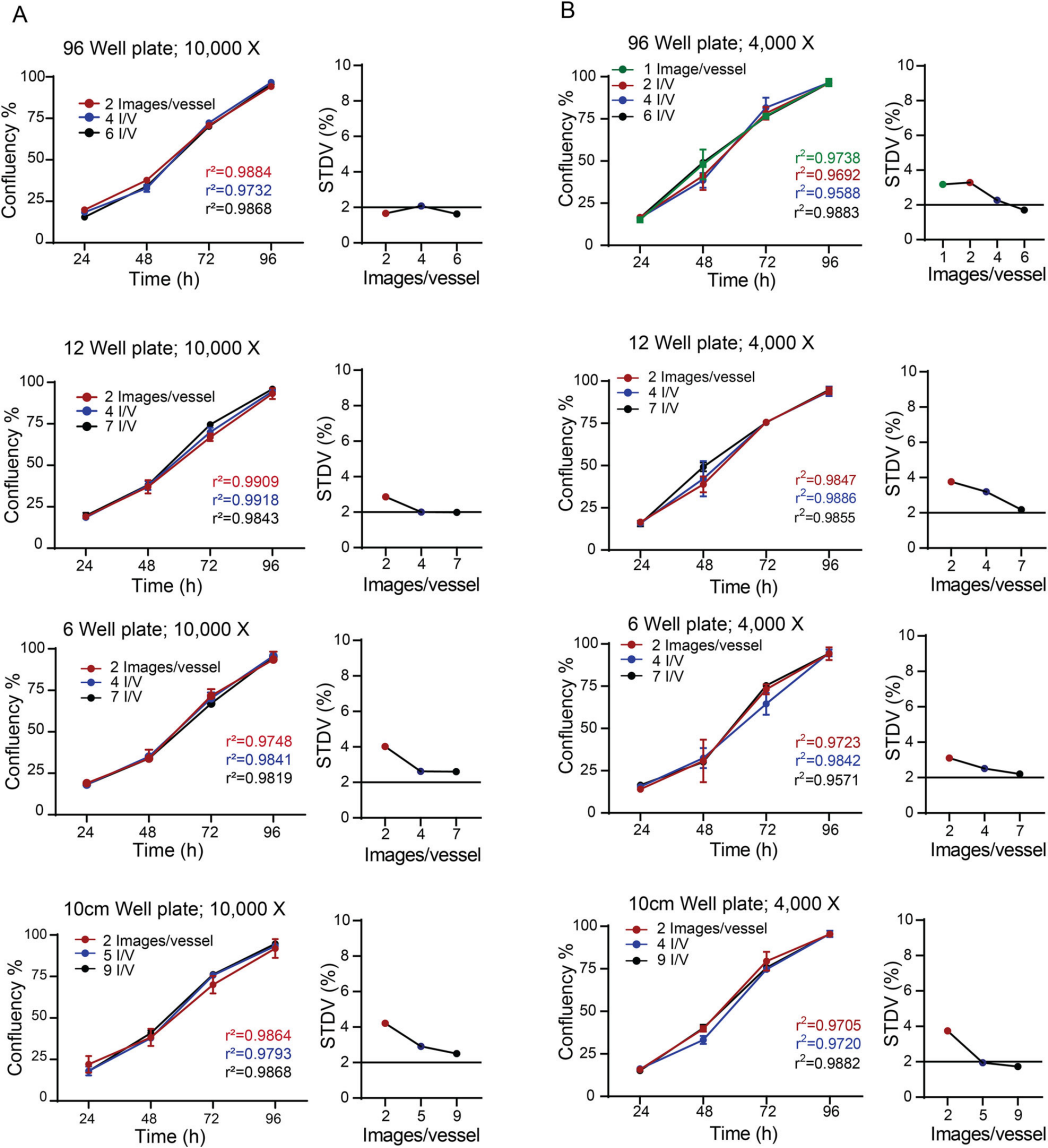
Optimizing image sampling for efficient and accurate confluency estimation in cell culture vessels at various magnifications. MCF7 cells were seeded in triplicate in 96, 12, 6-well plates and 10 cm dishes at 1.0E + 4 cells/well, 5.0E + 4 cells/well, 1.5E + 5 cells/well, and 1.5E + 6 cells/dish, respectively. Confluency of MCF7 cells in different culture vessel types was determined by Snapcyte™ Confluency at 24 h, 48 h, 72 h, and 96 h after seeding using Nikon ECLIPSE Ts2 inverted microscope at 10× (**A**) and 4× (**B**) magnifications, respectively. Numbers of images taken in each vessel type are shown in the corresponding panels. Experiments were repeated three times independently, and values are expressed as the mean. Standard deviation (STDV) of the values from three independent experiments was calculated and is shown in the right-side panels.

Since SnapCyte™ derives total vessel confluency from sampled images, an important question is how much of the vessel surface must be imaged to obtain an accurate estimate. We therefore quantified the fraction of each vessel type that needs to be captured to achieve stable confluency measurements. Using images acquired at 4000× and 10,000× magnification, we determined the minimum sampling area required to reach a standard deviation below 5%. For 96-well, 12-well, and 6-well plates, as well as 10 cm dishes, this corresponded to imaging approximately 36.5%, 3.0%, 0.6%, and 0.3% of the total vessel area at 10,000× magnification, and 22.8%, 18.9%, 13.8%, and 2.1% at 4000× magnification, respectively (Fig. 3A, B). The resulting number of images needed to capture these fractions was 2, 2, 4, and 5 per vessel.

The total imaging times for these vessels (all wells in a vessel) were 32, 4, 4, and 1 min, respectively. However, for 96-well plates at 4000× magnification, a single image covering 11.4% of the well was sufficient to accurately predict the confluency of the entire well (Fig. 3B, *upper panel*), reducing the total imaging time to approximately 16 min for the whole vessel. Although images were acquired manually, the low number of required images per vessel and the rapid analysis by the ML model make SnapCyte an efficient method for assessing confluency or density of cells in most basic laboratory settings. This efficiency could potentially be further enhanced by integration with an automated imaging system, offering even faster and more consistent results.

### Benchmarking SnapCyte™ confluency detection against standard proliferation assays

**Fig. 4:**
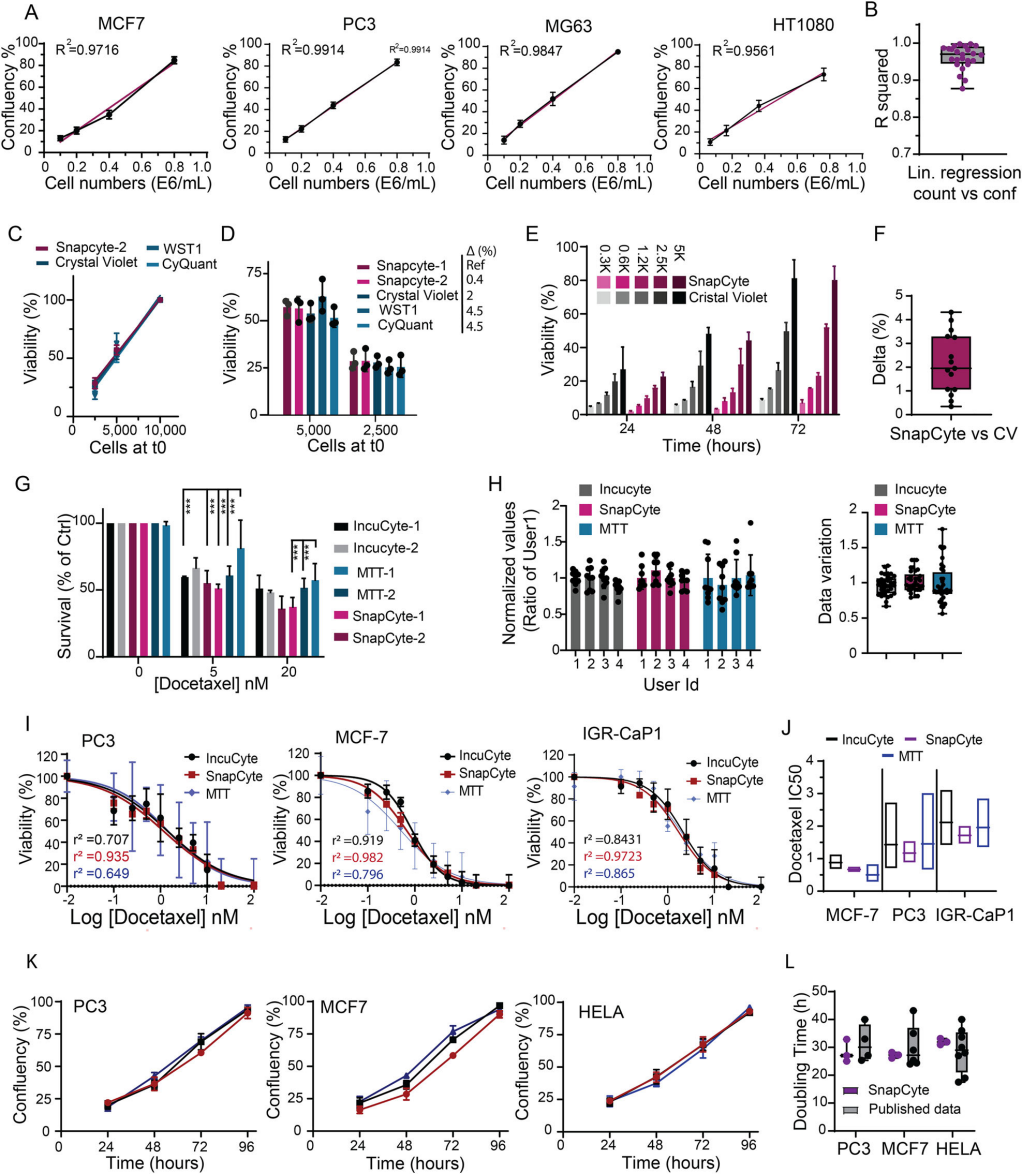
Benchmarking SnapCyte™ confluency detection against standard proliferation assays. **A** 1.0E + 5, 2.0E + 5, 4.0E + 5, and 8.0E + 5 MCF7 cells, PC3 cells, MG63 cells, and HT1080 cells were seeded in duplicate in 6-well plates. Confluency of cells was determined by SnapCyte™ at 6 h after seeding. Five images were taken per well at 4× magnification. Experiments were repeated three times independently, and values are expressed as the mean. Results of the first replicate are shown. Correlation between the confluency and cell counts was performed using linear regression analysis. The purple line represents the linear regression fit. R² is the correlation coefficient. **B** *R*² values of simple linear regression analysis from all individual experiments performed in (**A**) are plotted and shown. **C** Cells were seeded at three different concentrations, and cell viability was determined using SnapCyte™, Crystal Violet, WST-1, and CyQuant. Readouts were normalized to the highest cell count, and data were presented as a percentage. Statistical tests show no difference between the slopes (dF = 0.53, DFn = 4, DFd = 35, *P* = 0.71). **D** Normalized viability readouts comparison between SnapCyte™ and reagent-based assays from (**C**); no significant difference observed. **E** PC3 cells were seeded at increasing concentrations as shown in the panel. At 24 h, 48 h, and 72 h after seeding, cell viability was measured by SnapCyte™ Confluency and Crystal Violet separately. Experiments were repeated three times independently, and values are expressed as the mean. **F** Variability (delta) of normalized viability readouts comparison between SnapCyte™ and Crystal Violet. **G** PC3 cells were seeded in triplicate in two 96-well plates at 1.0E + 4 cells/well and were exposed to 0 nM, 5 nM, and 20 nM of Docetaxel for 72 h. Cell confluency was determined by IncuCyte®, SnapCyte™ Confluency, and MTT by two researchers separately (user 1 and user 2). Two images were taken per well using 4× magnification. Experiments were repeated three times independently, and values are expressed as the mean. **H** Four independent researchers (users 1, 2, 3, and 4) measured the viability of the same sample using three different techniques: IncuCyte®, SnapCyte™ Confluency, and MTT. Results were normalized to user 1 (Right panel). The data of four users were combined to show the standard error magnitude. **I** MCF7 cells, PC3 cells, and IGR-CaP1 cells were seeded in triplicate in 96-well plates at 1.0E + 4 cells/well. Docetaxel was diluted to 10 mM in DMSO. 24 h after cell seeding, 10 mM of Docetaxel in DMSO was diluted in the corresponding cell culture media to 0.1 nM, 0.25 nM, 0.5 nM, 1 nM, 2.5 nM, 5 nM, 10 nM, and 20 nM. Cells were exposed to increasing concentrations of Docetaxel for 72 h. Cell viability was determined by IncuCyte®, SnapCyte™ Confluency, and MTT. Two images were taken per well at 4× magnification. **J** Docetaxel IC50 value and confidence interval of all three assays. **K** MCF7 cells, PC3 cells, and HeLa cells were seeded in triplicate in 96-well plates at 1.0E + 4 cells/well. Confluency of cells was determined by SnapCyte™ Confluency at 24 h, 48 h, 72 h, and 96 h after seeding. Two images were taken per well at 4× magnification. Experiments were repeated three times independently, and values are expressed as the mean. **L** Doubling time of each cell line in each of the three independent experiments was calculated and is shown. Corresponding values reported in publications are also shown. Experiments were repeated three times independently, and values are expressed as the mean. *** (*p* < 0.0001).

Accurate and efficient monitoring of cell growth is essential for biological research and drug development studies. Traditional cell proliferation assays either track the number of cells over time or rely on surrogate readouts, such as metabolic activity, ATP, or the percentage of area covered by adherent cells. We aimed to investigate the correlation between confluency measurements by SnapCyte and cell counts. We first plated increasing concentrations of various cell lines, including epithelial (MCF7, PC3), mesenchymal (MG63), and fibrosarcoma (HT1080) cells, and assessed cell confluency 6 h after plating using SnapCyte. The correlation between cell counts and confluency was evaluated across three independent experiments. Results demonstrated a strong correlation between confluency assessed by SnapCyte and cell count, with a coefficient of determination (*R*²) greater than 0.9412 (Fig. 4A; Fig. S1A, C, E, G). Next, we performed a similar analysis 24 h after plating, where wells seeded with 1.00E + 06 cells had reached near-complete confluency (~100%). The data showed a high linear correlation (Fig. S1B, D, F, H). Linear regression analyses across all experiments, including four cell lines with three replicates at each time point, confirmed a robust correlation (average *R*² = 0.974) (Fig. 4B). Additionally, analysis of linear regression equations from replicates in each experiment revealed consistent slopes and interception points, underscoring the high reproducibility of the correlation (Fig. S1A–H). Collectively, these findings demonstrate that SnapCyte can use cell confluency to precisely report on cell numbers and proliferation rates in 2D cell cultures across diverse cell lines.

To further establish the performance and reliability of SnapCyte as a tool for precise monitoring of cell proliferation, we tested its accuracy and correlation in measuring cell growth across various techniques and assessed its performance in terms of ease of use, time efficiency, and reproducibility. We benchmarked SnapCyte against established reagent-based proliferation assays, including Crystal Violet (CV) staining, CyQuant, and WST-1. Initially, we evaluated the correlation between the normalized viability readouts from each assay and the initial cell counts at the time of seeding. We found a high correlation across all techniques with no significant statistical difference in the slopes between SnapCyte and the three alternative assays. Similarly, no significant difference was observed in SnapCyte data collected by two independent users (*F* = 0.53, dfn = 4, dfd = 35, *P* = 0.71) (Fig. 4C). We also assessed the variability (delta) of normalized viability readouts among the different methods. The delta between SnapCyte users was <0.5%, as compared to 2% for the colorimetric CV assay and 4.5% for the metabolic assays (WST-1 and CyQuant) (Fig. 4D).

In a subsequent experiment, we employed SnapCyte to monitor cell growth (measured as confluency over time) and compared these data against the conventional CV method (Fig. 4E). The data were similar with a delta value of 1.8% between the averages of the two methods (Fig. 4F). Additionally, no significant difference was observed between the two methods at any of the time points. To compare, SnapCyte provided fast (15 min/time point), accurate, and time-lapse growth data, facilitating direct use of the cell cultures in downstream applications. By contrast, the CV method necessitated multiple cultures for endpoint readouts and required more than 2 h of processing time.

We next evaluated the performance of SnapCyte as a readout for cytotoxicity relative to the live cell imaging system IncuCyte® and the MTT assay. In this study, we assessed the cytotoxic effects of docetaxel on PC3 cells at two different concentrations. The experiments were conducted in triplicate and repeated by independent users. Results demonstrated that SnapCyte-generated comparable values to both IncuCyte and the MTT assay, with SnapCyte and IncuCyte displaying lower standard deviations than the MTT assay (Fig. 4G). Additionally, SnapCyte exhibited less variability between independent experiments, further emphasizing its reliability and output reproducibility.

To measure inter-user reproducibility, the same biological condition was plated four times in nine replicates and measured by four experienced users using IncuCyte, SnapCyte, and MTT. The data indicated no difference between techniques and users with SnapCyte and IncuCyte (standard deviation 14% and 13%, respectively), and showed that these methods achieved less variability as compared to the MTT assay (27%) (Fig. 4H). This underscores the advantages of non-invasive measurement methods in providing consistent, user-independent data. Moreover, when the same experiment was measured by four different users setting their own parameters, paired measurements showed an average standard deviation of 6.5% and 7.5% for SnapCyte and IncuCyte, respectively.

To validate the performance of SnapCyte in drug testing applications, we evaluated the half maximal inhibitory concentration (IC50) of docetaxel across three distinct cell lines: MCF7, PC3, and IGR-CaP1. Each cell line was tested in three independent experimental setups, and the IC50 values were calculated using transformation and sigmoidal regression analyses. SnapCyte demonstrated robust correlation coefficients for PC3 (0.935), MCF7 (0.982), and IGR-CaP1 (0.972), with IC50 confidence intervals of [0.9–1.5], [0.59–0.75], and [1.5–2], respectively. These values were compared to those obtained with IncuCyte and the MTT assay, which generated lower correlation coefficients and wider IC50 confidence intervals of 0.707 [0.7–2.7], 0.919 [0.68–1.13], 0.843 [1.4–3.1] for IncuCyte and 0.649 [0.7–3], 0.761 [0.24–0.74], 0.865 [1.35–2.85] for the MTT assay in the respective cell lines (Fig. 4I, J).

Lastly, we evaluated the SnapCyte model’s ability to replicate published data by determining the doubling time of three cell lines across three independent experiments. Our results not only aligned closely with each cell line’s published doubling time, but also showed that the doubling time values were near the median of values from independent studies (Fig. 4K, L).

Combined, the results indicate that SnapCyte provides highly accurate and reproducible measurements of cell growth and confluency across diverse cell lines. The system’s non-invasive and time-lapsed capabilities make it an efficient and reliable alternative to conventional instrument and reagent-based assays. SnapCyte offers comparable accuracy, reduced variability, and faster processing times and is well-suited for routine growth and survival assessments in cell biology laboratories.

### Optimization of deep learning models for cell counting and viability assessment

**Fig. 5:**
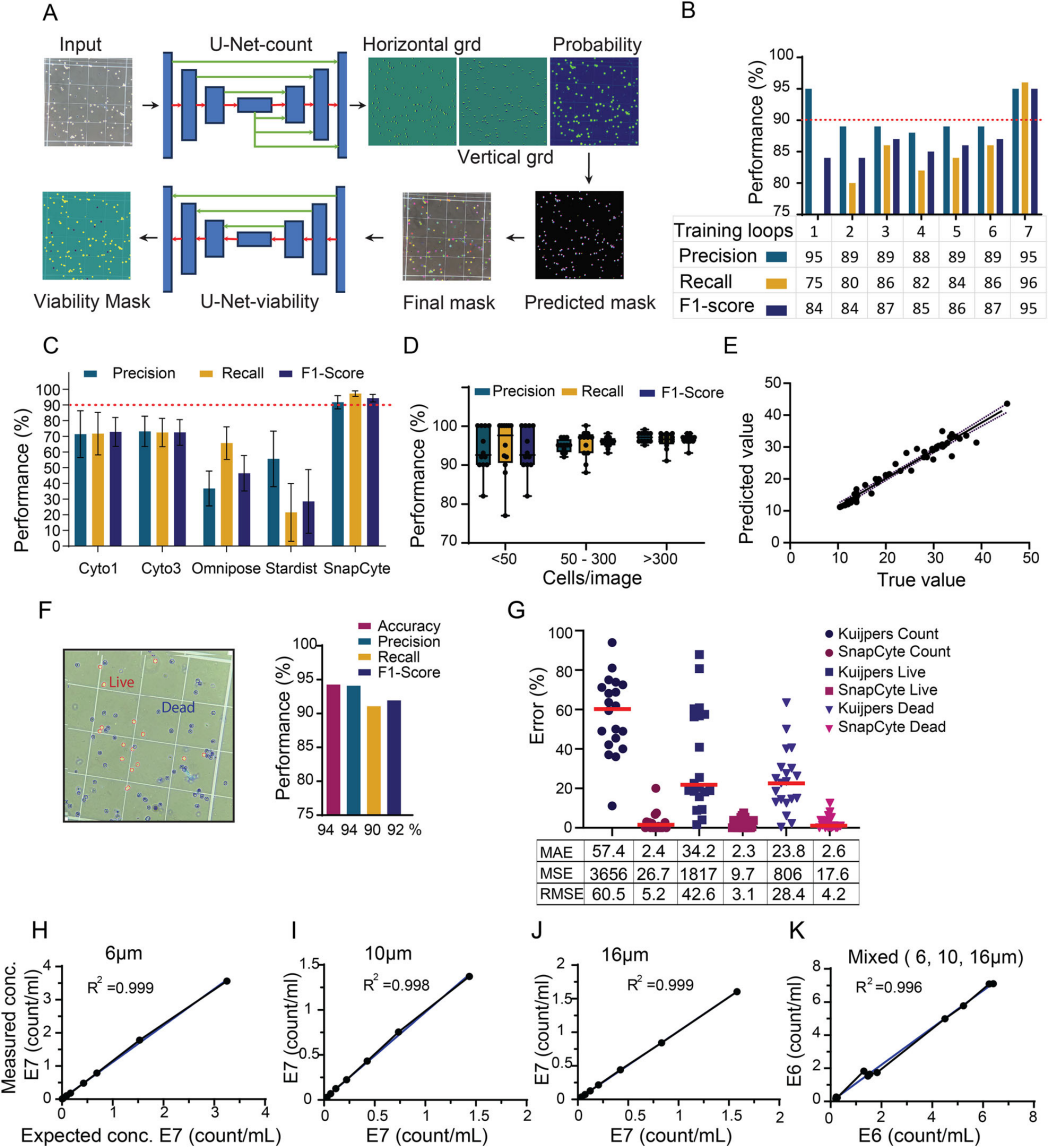
Optimization of deep learning models for cell counting and viability. **A** Architecture of the SnapCyte cell count and viability pipeline. The Cellpose-based neural network was fine-tuned to predict cell probability maps together with horizontal and vertical gradient (flow) matrices. These outputs are combined and post-processed to generate final instance segmentation masks. The identified objects are subsequently processed through a U-Net–based classifier to determine cell viability (live/dead discrimination). The representative image shown corresponds to PC3 cells stained with Trypan Blue for viability assessment. The model was trained on a large and diverse dataset comprising multiple cell lines, peripheral blood mononuclear cells (PBMCs), red blood cells (RBCs), and size reference beads. Segmentation is performed on the brightfield image independently of color intensity thresholding, and viability classification is applied only after object-level segmentation. **B** Performance of the cell counting model across seven iterative human-in-the-loop training cycles. Precision, recall, and F1 score are shown for each iteration, with a target threshold of ≥95% achieved across all metrics. **C** Comparison of precision, recall, and F1 score for Cyto1, Cyto3, Omnipose, StarDist, and SnapCyte models for cell segmentation and cell counting on a dataset of 50 images. SnapCyte demonstrates superior performance, achieving the highest scores across all three metrics. Error bars indicate standard deviations across test samples. **D** Performance test on images containing a different range of cell counts by region of interest (<50, 50–300, and >300 cells/ ROI; hemocytometer 1 × 1 mm square). **E** Correlation between the manual mask object size and the cell count model size estimation, showing a high correlation and accuracy, dashed lines represent error bars. **F** Representative example of cell count (PC3 cell line) and viability detection masks produced by SnapCyte (left). Quantitative performance metrics for the viability classification model are shown on the right. **G** Comparison of error distributions for total cell count, live cell identification, and dead cell identification between the Kuijpers model and SnapCyte. Scatter plots show individual sample errors, with red bars indicating mean error for each category. Mean Absolute Error (MAE), Mean Squared Error (MSE), and Root Mean Squared Error (RMSE) are summarized in the table below. Validation of SnapCyte cell counting using serial dilutions of monodisperse beads with diameters of 6 µm (**H**), 10 µm (**I**), and 16 µm (**J**). Beads were diluted in distilled water, loaded onto a hemocytometer, and counted using SnapCyte. Expected versus measured bead concentrations are shown with corresponding correlation coefficients. **K** In addition, three sizes of beads were mixed in a 1:1:1 ratio at three concentrations (5.0E + 6, 1.25E + 6, and 2.5E + 5). 10 µl of beads were loaded on a hemocytometer, and the bead numbers were determined by SnapCyte™ Count. Four images were taken per sample. In parallel, the number of beads was determined manually. Comparison between the results of SnapCyte™ Count and manual counting was performed using linear regression. *R*² is the correlation coefficient.

Accurate cell counts are essential for cell biology assays, particularly for non-adherent cells where confluency measurements are inadequate. We next aimed to optimize an ML model for assessing cell numbers, employing the Cellpose model [32], a general U-Net architecture pretrained on a diverse range of cellular images (Fig. 5A). We used various cell lines, beads, PBMCs, and RBCs that were loaded onto a hemocytometer to acquire images for annotation. The dataset included images obtained with and without cell death dyes (Trypan Blue and Erythrosin B) to better represent real-world laboratory conditions.

Training and fine-tuning the Cell Count model using our human-in-the-loop approach (Fig. 1A) required seven iterative cycles to achieve Precision and Recall rates of over 95% in detecting cells while excluding debris (Fig. 5B). However, further improvements posed challenges due to a 5% variation in labeling, which may result from discrepancies even among cell annotation experts. A drop in the F1 score during cycle 4 was attributed to the introduction of a more complex and variable dataset, reflecting more realistic scenarios. After cycle 6, our analysis indicated that annotation inconsistencies were the primary obstacle for further improvements, as supported by previous research by Kang et al. [54]. A dataset review identified and resolved these inconsistencies, leading to improved performance in the final cycle, achieving 95% Precision and Recall at cycle 7.

We further evaluated the performance of SnapCyte in segmenting and counting single cells against other state-of-the-art models (Cyto1 [32], Cyto3 [55], Omnipose [56], and StarDis [31]) on a randomly selected dataset of 50 images. The results reveal that SnapCyte consistently outperforms the alternatives across all three key metrics: precision, recall, and F1 score (Fig.5C). SnapCyte achieves the highest average precision of 94%, significantly surpassing Cyto1 and Cyto3 (both at approximately 75%), and far exceeding Omnipose (37%) and StarDist (48%). Similarly, the recall of SnapCyte is 97%, considerably higher than Cyto1 (72%) and Cyto3 (78%), with Omnipose (63%) and StarDist (42%) demonstrating inferior performance. Representative raw images together with their corresponding segmentation masks and quantitative outputs used for this comparison are provided in the Supplementary Materials (Raw Data, Fig. 5C), allowing independent visual inspection of model performance. Finally, SnapCyte achieves the highest F1 score at 95%, reflecting its superior balance between precision and recall, compared to Cyto1 (73%), Cyto3 (74%), Omnipose (49%), and StarDist (45%). Among the tested models, Cyto1 and Cyto3 demonstrate moderate and consistent performance across all metrics, though they lag behind SnapCyte. This is expected as the Cyto1 and 3 are general models, while SnapCyte is specifically fine-tuned on cell culture microscopy images. Omnipose exhibits substantial deficiencies in precision and F1 score, likely due to its reduced ability to accurately detect individual cells. StarDist shows poor overall performance in this diverse dataset, with significantly low precision and recall, indicating challenges in both cell detection and segmentation. These results highlight the robustness and accuracy of SnapCyte, establishing SnapCyte as the most reliable tool for cell counting among the models tested.

To assess model performance across various cell densities, we evaluated Precision, Recall, and F1 scores using subsets of our dataset compiled based on annotated cell numbers per image. We analyzed 12 images with fewer than 50 cells, 12 images with 50–300 cells, and 12 images with more than 300 cells. The data showed consistent and strong performance across all groups (Fig. 5D). As the number of cells increased, the metrics (Precision, Recall, and F1) improved, reaching 0.95 for images with more than 50 cells. Although we did not determine an upper limit for cell numbers, the model’s robustness in denser images suggests its suitability for applications involving cell clumping and overlap.

Given the importance of precise cell size measurements in biological assays, particularly for distinguishing cell types, we optimized a Size Estimation model, which works in conjunction with the Cell Count model. The process involves two steps: first, the Cell Count model evaluates images using a default diameter to compute a Style array—256 float values array, representing image features. A linear regression model then predicts cell size based on the aforementioned Style array. Finally, the Cell Count model generates output masks using the median of the cell sizes, as determined by the Size Estimation model. The image is resized according to the predicted diameter, and the Cell Count model generates output masks, from which the median object size is determined as the final predicted size. Utilizing a visual representation where the x-axis represents the true cell diameter and the y-axis the predicted cell diameter (in pixels), we observed a close alignment between predicted and actual sizes (*R*² = 0.95) (Fig. 5E). This strong correlation confirms the accuracy of the Size Estimation model, making it a reliable tool for downstream applications requiring precise cell size measurements. The combination of robust predictions from the Cell Count model and linear regression ensures accurate size estimations with low error rates suitable for most cell counting requirements.

In many cell biology assays, cell count is meaningful only when combined with viability assessments. Thus, we aimed to develop an ML model capable of distinguishing live and dead cells based on Trypan Blue or Erythrosin-B staining, two commonly used dyes for cell viability assessment [57]. To determine cell viability, images of cells stained with these dyes first undergo analysis by the Cell Count model to identify individual cells. The resulting segmentation mask, combined with the original image, is then processed through our Viability model. Evaluation metrics, including Accuracy, Precision, Recall, and F1 score, are depicted in (Fig. 5F), alongside an example output image demonstrating the accuracy of the viability detection.

Among the available machine learning models for cell counting and segmentation, to our knowledge, only the model published by Kuijpers et al. [18] was reported to distinguish live and dead cells using trypan blue staining. Using a dataset of 20 images of trypan blue-stained cells loaded into different types of chambers (e.g., hemocytometers and KOVA slides), we compared SnapCyte to the Kuijpers model for total cell count and live/dead cell discrimination. For total cell count, Kuijpers exhibited high error rates (MAE: 57.4%, MSE: 3656, RMSE: 60.5%), indicating consistently large errors, with occasional extreme outliers contributing to the higher RMSE. In contrast, SnapCyte achieved significantly lower errors (MAE: 2.4%, MSE: 26.7, RMSE: 5.2%), demonstrating both accuracy and consistency. Similarly, for live and dead cell classification, SnapCyte maintained minimal errors (MAE: 2.3–2.6%, RMSE: 3.1–4.2%), compared to Kuijpers’ higher errors (MAE: 34.2–23.8%, RMSE: 42.6–28.4%) (Fig. 5G). Although the Kuijpers model had false positive and false negative rates below 2% for live/dead classification, its higher overall errors stemmed from poor cell segmentation, highlighting the importance of accurate segmentation. The low and closely aligned MAE and RMSE across all tasks reflect precise and reliable predictions and confirm the robustness and superior performance of SnapCyte in cell analyses.

Accurate detection and counting of particles of different sizes is critical when working with mixed populations of cells or particles, such as those commonly used in co-cultures, primary cultures, and patient samples. To ensure that our model could handle diverse sample types, we tested its capability to count shapes of different sizes. We used 6 µm, 10 µm, and 16 µm flow cytometer size reference beads, which were diluted to different concentrations or mixed in a 1:1:1 ratio. The number of beads in each sample was compared between SnapCyte and manual counting. Our results showed a strong correlation between the SnapCyte values and expected concentrations for all three bead sizes, both separately and when mixed together (Fig. 5H–K). Specifically, the coefficient of determination for the 16 µm beads was as high as 0.9999 and >0.99 for 6 µm and 10 µm beads. This indicates that our model can accurately detect and count homogeneous and heterogeneous samples with different particle sizes, highlighting its robustness in handling mixed cell populations (Fig. 5K).

### Comparative performance of the SnapCyte™ cell count model and conventional cell counting methods

**Fig. 6:**
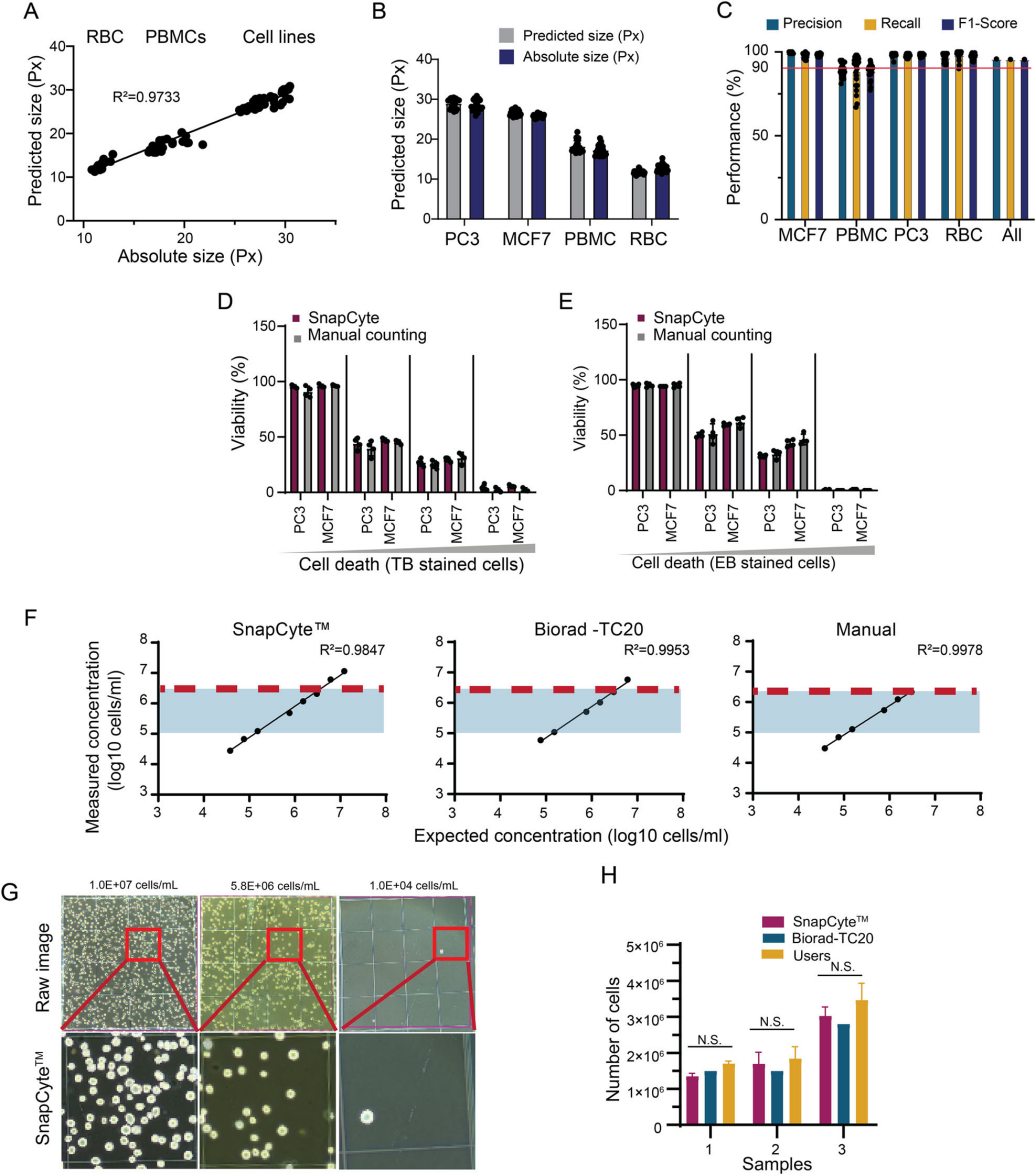
Comparative performance of the SnapCyte™ cell count model against conventional cell counting methods. **A** Sizes (in pixels) of cells (RBC, PBMCs, and cell lines) in 96 images were determined manually by scientists (absolute size) and by SnapCyte™ Count (predicted size) separately. Data show a high correlation between the two methods in the entire dataset using linear regression; correlation coefficient *R*² = 0.9733. **B** Comparison of the size estimation of (**A**) in each cell type separately. No statistical significance was observed. **C** Cell count model validation parameters. **D** PC3 or MCF7 live cells were mixed with dead cells at different ratios and stained with Trypan blue or **E** Erythrosin B. Using a hemocytometer, images were analyzed by SnapCyte™ and manually by an expert. The percentages of viable cells determined by both methods were compared; no significant difference was present between the two methods. **F** PC3 cell pellets were collected, and then resuspended in DMEM supplemented with 10% FBS in decreasing concentrations: 1.0E + 7 cells/mL, 5.8E + 6 cells/mL, 2.25E + 6 cells/mL, 1.03E + 6 cells/mL, 5.4E + 5 cells/mL, 1.1E + 5 cells/mL, 5.7E + 4 cells/mL, and 1.0E + 4 cells/mL. 10 uL of each sample was individually loaded on a Hausser ScientificBright-Line™ Phase Hemacytometer, and the cell numbers were determined by Snapcyte™ Count. Four images were taken per sample. In parallel, 10 uL of each sample was individually loaded on cell counting slides, and the cell numbers were determined by the TC20 Automated Cell Counter. In addition, cell numbers of each sample were also determined manually by an experienced user of hemocytometers using benchtop counters. **G** Representative images showing Snapcyte™ Count software detecting cells at different concentrations. **H** MCF7 cells were diluted in 3 different concentrations. For each concentration, 10 uL of sample was mixed with 10 uL of Trypan Blue Solution, 0.4%. 10 uL of the solution of the three samples was individually loaded on three Hausser Scientific™ Bright-Line™ Phase Hemacytometers, and the cell viability was determined by Snapcyte™ Count + viability module. Four images were taken per sample. In parallel, 10 uL of each sample was loaded on cell counting slides, and the cell numbers were determined by the TC20 Automated Cell Counter. In addition, cell numbers and the viability of the cells in three samples were also determined manually by four experienced users of hemocytometers using benchtop counters. Experiments were repeated three times independently, and values are expressed as the mean. N.S.: Not significant. (*p* > 0.05).

We first tested the SnapCyte size estimation model using a diverse set of biologically relevant samples. A dataset of 96 images of human red blood cells (RBCs), human peripheral blood mononuclear cells (PBMCs), PC3, and MCF7 cells was employed. The predicted values of SnapCyte were comparable to absolute cell sizes determined by scientists (Fig. 6A). The results demonstrated a strong correlation between SnapCyte and the actual cell sizes for all three types of samples (*R*² = 0.9733) (Fig. 6B). Furthermore, the Accuracy, Recall, and F1 score of the model were consistently above 90%, regardless of the cell type analyzed (Fig. 6C). These findings highlight the ability of SnapCyte to accurately determine and differentiate cell sizes across various sample types, including heterogeneous PBMC samples.

Next, we benchmarked the SnapCyte model’s counting and viability capabilities against manual assessments performed by experienced scientists. We used a test dataset of 128 images featuring Trypan Blue- or Erythrosin-B-stained PC3 and MCF7 cells with varying viability percentages. Cells were loaded onto hemocytometers or KOVA slides for evaluation by the SnapCyte viability module. Results showed that the average absolute difference between SnapCyte and manual counting was less than 5% for both Trypan Blue and Erythrosin-B staining across different live/dead cell ratios (Fig. 6D, E). Importantly, the model’s performance was independent of the slide type when tested on KOVA slides (Fig. S2). This demonstrates that the SnapCyte viability model is a reliable tool for quantitative microscopy, providing accurate viability measurements for cells stained with two commonly used viability dyes.

Most automated cell counters have a reliable detection range of 1.00E + 05 to ~1.00E + 07 cells/mL [58]. This range limitation sometimes requires cells to be concentrated or diluted to obtain accurate counts. We tested the SnapCyte model’s performance across a wide range of cell concentrations to establish the dynamic range. Data showed that SnapCyte provided reliable cell counting in the range of 1.00E + 04 cells/mL to 2.50E + 07 cells/mL (Fig. 6F), as indicated by a high coefficient of determination (*R*² = 0.9847), comparable to Bio-Rad (*R*² = 0.9953) and manual counting (*R*² = 0.9978). We further assessed the ability of SnapCyte to count cells in serially diluted PC3 cell samples. High-resolution images revealed that each cell was effectively segmented even in the highest concentration of 2.50E + 07 cells/mL (Fig. 6G). This demonstrates that SnapCyte accurately distinguishes individual cells in densely packed samples that are difficult to count in manual settings.

Finally, we evaluated the reproducibility of SnapCyte compared to manual counting. Four researchers manually counted MCF7 cells at three concentrations (5E + 06 cells/mL, 1.6E + 06 cells/mL, and 2.9E + 06 cells/mL) using a hemocytometer. In parallel, each researcher determined the cell count using SnapCyte. As a reference, cell counts were also measured using the TC20 cell counter. SnapCyte results showed no statistically significant differences between SnapCyte and manual counting measurements. It also showed smaller standard error deviations for SnapCyte measurements compared to manual counting (Fig. 6H).

Altogether, our data demonstrate that SnapCyte provides a robust, accurate, and efficient ML-based solution for assessing cell confluency, counting, and viability across diverse cell types and experimental conditions. Its U-Net architecture achieves >90% accuracy for confluency detection, with strong reproducibility and minimal deviations under varying image qualities and densities. SnapCyte also demonstrated high correlation with traditional proliferation assays and outperformed manual counting methods, extending its dynamic range to 2.50E + 07 cells/mL. The system’s non-invasive, time-efficient, and highly reproducible measurements make it a reliable alternative to conventional assays, offering significant advantages for routine cell biology workflows and drug development studies.

## Discussion

Scientists face numerous challenges related to basic cell analytics proficiency. Research costs have increased drastically, the demand for faster methods has intensified, and the scientific community has encountered a reproducibility and replicability crisis [59], which reflects a multifactorial phenomenon where standardization and accuracy of technologies play significant roles.

In cell analytics, traditional methods, such as manual cell counting and reagent-based approaches, are time-consuming, prone to human error, and require costly instrumentation. Additionally, recent image processing technologies have demonstrated limitations, including being time-consuming and requiring users to define subjective parameters (e.g., ImageJ) or being inaccessible to many research groups due to cost. There is a critical need in cell biology research to eliminate user variability inherent in conventional methods and to develop accessible and reliable solutions to ensure data comparability between studies. AI-based systems have emerged as transformative tools in cell analytics, promising rapid, unbiased, and consistent cell analysis readouts. Recent advancements in automation and ML for basic cell culture tasks have shown promise, but only a few have proven to be accurate and applicable to a wide range of cells and experimental conditions. Many ML models currently available are trained on open-source datasets that feature idealized, clean images, often lacking the variability encountered in everyday cell culture environments. These datasets typically do not reflect the real-world challenges, such as debris, clumps, non-uniform lighting, or irregularities in sample preparation, making their models less effective in practical applications. Consequently, there is still an imminent demand for accurate and affordable deep learning models to be widely deployed in research.

SnapCyte was developed using a rigorously curated dataset encompassing a wide array of cell lines and conditions, meticulously annotated to customize AI models for the detection and analysis of cells across diverse experimental setups. We have customized three ML models to measure cell confluency, cell count, and cell viability—three readouts that constitute the basics of cell analytics in a cell biology or life science laboratory. SnapCyte achieves high precision and recall for all three models across all cell lines and image qualities that were tested, reflected by high F1 scores. Notably, for the Cell Count ML model, accuracy increased in samples containing a higher number of cells, proving the superiority of this method in counting concentrated samples. This is important, as dilution affects the prediction accuracy of total cell counts [21].

SnapCyte consistently outperforms existing publicly available models with exceptionally low error metrics, demonstrating high precision and reliability. While some models achieve a balance of accuracy and consistency, others exhibit significant errors across all metrics, indicating a need for substantial adjustments or alternative modeling approaches to achieve acceptable predictive accuracy. This highlights the importance of customization in machine learning models. Generalist models like Cellpose are designed for broad applications, which can limit their precision, whereas SnapCyte was developed using specialized and diverse datasets, ensuring superior accuracy and performance for cell culture settings.

Confluency measurements are crucial for ensuring the consistency and reliability of cell culture experiments. Traditional methods often rely on subjective visual assessments, leading to significant errors and variability. Studies have shown that confluency eyeballing errors can be very high, especially at higher confluency levels, impacting experimental outcome and reproducibility. For example, a study published by Lin et al. discusses the challenges in accurately determining cell confluency, highlighting that visual estimations can greatly vary among researchers, especially among those with less experience. This variability can exceed 30%, affecting the consistency of experimental results [60]. High confluency levels can lead to contact inhibition, altering gene expression involved in cell cycle regulation and apoptosis, thus affecting studies on cell growth, migration, and response to treatments. Our validation tests of the confluency model on independent datasets, which included a high number of cell lines and random images from publications, demonstrated the universality of the ML model and its applicability to a wide range of laboratory settings (lighting, image resolution, and cell models). The F1 score remained above 91%, and the average deviation from manual masks was 3%.

The SnapCyte confluency model is highly relevant for data standardization in cell biology research. It enables scientists to instantly evaluate seeding homogeneity and report it accurately. This is crucial for experiments that are sensitive to seeding variation, such as genome editing and differentiation studies, thereby enhancing replicability and reproducibility. This model also assists scientists in maintaining healthy cell cultures by ensuring that cells are seeded homogeneously and passaged at adequate confluency to prevent genetic drift in cell cultures.

Additionally, SnapCyte allows for non-invasive, time-lapse cell growth measurement from the same vessel, enabling scientists to determine the optimal timing for treatments or cell editing. This capability helps avoid potential issues related to over- or under-seeding, which can significantly affect experimental outcomes.

Balancing the positive considerations, it is important to acknowledge the limitations of image-based analysis, particularly when based on cell confluency. In some conditions, treatments or cell manipulations may lead to morphological changes and alterations in cell attachment, which can affect cell size and confound confluency measurements. This could result in cell growth assessment errors. Nevertheless, because the analysis is image-based, scientists can visually inspect cell morphology and detect changes, thereby mitigating the risk of misleading information.

Accurate cell counting is equally critical in cell biology research. Manual counting methods are prone to significant errors, while newer automated counters struggle with complex samples containing cells of different sizes. SnapCyte addresses these challenges by accurately counting particles of various sizes, as demonstrated in correlation studies with manual counting (*r* > 0.99). Notably, this model can count heterogeneous samples containing beads of varying sizes (6, 10, and 16 μm), further validated by its ability to distinguish different sizes accurately, including distinct populations of RBCs, PBMCs, and cancer cell lines. The superior performance of SnapCyte relative to general-purpose segmentation models likely reflects disparities in training dataset composition and task specificity. Many such models are trained on heterogeneous image collections with few, if any, hemocytometer images and limited exposure to the structured background patterns characteristic of counting chambers. Hemocytometer images present specific challenges, including grid lines, debris, staining variability, and out-of-focus cells, which can confound models not explicitly trained on such data. In contrast, the SnapCyte models were trained on curated, task-specific datasets enriched for hemocytometer and real-world cell culture images, enabling improved robustness under these conditions. Additionally, SnapCyte exhibits exceptional accuracy in detecting cells stained with Trypan Blue or Erythrosine B to assess viability. As many laboratories transition away from Trypan Blue staining due to its toxicity, the ability to use Erythrosine B provides a safer alternative. This versatility makes SnapCyte an impactful tool for measuring cell viability in complex biological samples.

Since SnapCyte uses images to analyze cells, an important consideration is how representative the images are of the entire vessel, especially in the context of cell proliferation. Like many other techniques in cell culture data analysis, cell confluency is based on image sampling, and in the case of adherent cells, the homogeneity of the culture is a cornerstone for performing accurate and reproducible data analysis. Assuming homogeneous seeding, SnapCyte requires a minimal sampling number (i.e., 1–2 for a 96-well plate, 2–4 for 6- and 12-well plates, and a 10 cm vessel). This ensures that SnapCyte analyses are fast and return data within a few minutes if using manual imaging. The ability of SnapCyte to process data within minutes without necessitating invasive procedures aligns with the pressing need for versatile analytical tools that are compatible with downstream processes.

We also investigated whether the cell confluency readout from SnapCyte could be used as a surrogate readout for cell proliferation and cytotoxicity assays. First, the correlation between cell counts and SnapCyte confluency was >0.95 in all conditions tested, indicating that confluency could predict cell number and, hence, proliferation (Fig. 4A and Fig. S1). When compared to reagent-based assays, results were comparable to standard methods with a low delta variation. When compared to Crystal Violet, the most commonly used manual assay for confluency, the variation was lower than 4% in all conditions. This offers the added advantage of avoiding errors related to Crystal Violet staining steps and reducing seeding-related variability between vessels by acquiring data from the same vessel. This approach also reduces the use of toxic reagents and saves time by allowing immediate use of cells in downstream assays.

Compared to the IncuCyte®, data obtained on SnapCyte™ were similarly accurate. Also, data between different SnapCyte users was more consistent as parameter settings were predefined. Furthermore, when compared to MTT and IncuCyte, IC50 values of cell lines treated with docetaxel and calculated with SnapCyte were more accurate, as evidenced by a high non-sigmoidal correlation coefficient (Fig. 4I). As such, SnapCyte produced similar IC50 values as other techniques but with smaller confidence intervals. While SnapCyte is not tied to an automation system, manual image acquisition remained within acceptable time limits (15 min). SnapCyte allows acquisition from any type of vessel and microscope, whereas many other methods and techniques are tied to their own instruments (e.g., IncuCyte). Moreover, the phone app version of SnapCyte transforms a smartphone into a cell analytics platform that can perform instant analyses on the device. Additionally, SnapCyte reduces contamination risks by eliminating the need to transfer cells between different rooms for measurement, maintaining the integrity of cell cultures and experimental conditions. Finally, the last validation involved comparing SnapCyte-generated data with published values of doubling time, demonstrating the accuracy of SnapCyte by producing values close to the median and demonstrating highly reproducible results.

For the cell count readout, SnapCyte uses a hemocytometer, but it can also be used with any type of slide. It eliminates the need for additional machines and specific slides. SnapCyte has a wide dynamic range, allowing quantification of highly concentrated samples and different sizes of cells. The data from SnapCyte is user-independent, and the variation is minimal. Importantly, SnapCyte performs segmentation without user-defined parameter tuning or threshold adjustment, eliminating operator-dependent variability and ensuring fully automated and reproducible analysis across experiments.

A major limitation in current cell imaging analysis workflows is their dependence on user expertise, manual parameter tuning, and assay-specific optimization, which limits reproducibility and scalability across laboratories. SnapCyte addresses this gap by providing a user-independent, parameter-free analysis framework that reproduces expert-level performance without requiring image preprocessing, threshold adjustment, or manual intervention. SnapCyte is hardware-agnostic and operates on standard brightfield images acquired using commonly available laboratory equipment, enabling broad adoption without additional capital investment. This facilitates seamless integration into existing workflows across academic and industrial settings.

A key contributor to SnapCyte’s robustness is the task-specific training strategy. Rather than relying on generic image datasets, models are trained on curated data reflecting real cell culture conditions, including variability in morphology, staining, debris, grid artifacts, and imaging quality. This enables reliable performance in both cell confluency and cell counting/viability applications, where general-purpose models often fail.

In this study, we outline how scientists can use SnapCyte to extract multiple quantitative readouts from the same experimental data image, including cell confluency, proliferation rates, doubling time, EC50 values, and viability, without changing analysis tools or workflows. By reducing analytical complexity and user dependence, SnapCyte supports standardization and democratization of quantitative cell imaging, empowering researchers to generate reproducible, high-quality data regardless of computational literacy.

## Materials and methods

### Model architecture

In this study, we evaluated multiple deep learning architectures to determine their suitability for cell detection and segmentation tasks. Models such as U-Net, Mask R-CNN, and YOLO were tested for their ability to identify cellular features in microscopy images. Initial evaluations revealed that baseline U-Net and Mask R-CNN faced challenges in accurately detecting specific features in complex microscopy data. While YOLO showed efficiency in fast object detection, its precision was inadequate for the requirements of life science imaging [29]. Nevertheless, U-Net emerged as the most effective for cell microscopy, owing to its use of skip connections and its symmetrical structure, which improved feature extraction and boundary delineation [61]. Hence, our Confluency model uses a standard U-Net with a 1024 × 1024 × 1 input. The contracting path comprises four blocks: each block has two 3 × 3 convolutions with 64, 128, 256, and 512 filters, respectively, followed by 2 × 2 max pooling; a 0.5 dropout is applied after the fourth block. The bottleneck consists of two 3 × 3 convolutions with 1024 filters and a 0.5 dropout. In the expansive path, we perform 2 × 2 upsampling followed by a 2 × 2 convolution (512 → 256 → 128 → 64 filters, ReLU, He-normal), concatenate with the corresponding feature map from the contracting path, and apply two 3 × 3 convolutions at each level. A final 3 × 3 convolution with two filters (ReLU) and a 1 × 1 convolution with sigmoid activation produces the binary confluency mask. Each segment of the architecture is critical for capturing features at different resolutions and effectively combining them for precise pixel-wise classification [33]. The network is compiled with the Adam optimizer (LR 1e-4) and binary cross-entropy loss.

We tested several Count models, including those utilized in literature and competition datasets, such as those from Kaggle, which often required specific image types (e.g., grayscale or single-cell fluorescent images). For instance, DeepCell [62] was designed for single-cell fluorescent microscopy images, while CellProfiler [63] underperformed on our multi-cell culture images, likely due to its instrument-specific limitations. DeepLab demonstrated promising results in general semantic segmentation tasks.

Finally, we adopted the Cellpose architecture (for architectural details, see [32]) and fine-tuned it on our dataset. Although pretrained Cellpose offers robust, general-purpose cell segmentation, additional training enhanced its precision for our specific cell counting and segmentation tasks across varied imaging conditions.

We performed a comprehensive evaluation of several regression models for cell size estimation, including Support Vector Regressor, Elastic Net Regressor, K-Nearest Neighbors Regressor, Random Forest Regressor, Gradient Boosting Regressor, and Linear Regressor. We conducted experiments using our dataset of images and compared the Mean Squared Error (MSE) across all models, yielding values of 43.5, 40.1, 36.1, 23.6, 12, and 10.9, respectively. Our analysis, based on these experiments, revealed that Linear Regression exhibited greater robustness when applied to unseen images from our dataset, outperforming the other models. Building upon Cellpose’s Linear Regression Size Model [32], we integrated this Linear Regression model into our pipeline for cell size estimation. This addition ensures precise determination of cell diameter size, thereby enhancing the reliability and performance of our cell counting system across a wide range of imaging conditions.

In our efforts to develop an optimal solution for detecting cell viability, we initially utilized unsupervised ML clustering algorithms to differentiate between live and dead cells in cell culture images. Unsupervised learning, beneficial in contexts without explicitly labeled data, allows algorithms to independently identify patterns or clusters within the data. However, these algorithms often classify images into two clusters, even when only live or dead cells are present. Among the unsupervised algorithms tested, K-means demonstrated acceptable performance with Trypan Blue-stained images but was less effective with other stains, such as Erythrosin B. Consequently, we adopted a supervised U-Net variant for viability detection. This U-Net variant begins with four 3 × 3 convolutional layers (two of which are each followed by 2 × 2 max pooling) using ReLU activations for feature extraction. Three 2 × 2 transposed convolutional layers then upsample the features for spatial reconstruction. A final 1 × 1 convolution with Tanh activation produces a single-channel viability mask. Ultimately, we trained our Viability model, achieving satisfactory performance across various types of images. Segmentation and viability classification are implemented as two independent steps; cell segmentation is performed using morphological methods without reliance on dye color intensity, and viability assessment is applied only after object-level segmentation. Staining information remains part of the input image, and the viability classifier is trained to consistently apply the same visual criteria used in manual hemocytometer-based live/dead scoring, thereby standardizing operator interpretation rather than bypassing it.

### Datasets

24 adapter and a variety of cell phones (iOS and Android systems) and microscopes (Nikon ECLIPSE Ts2 inverted microscope, Leica DM1000 LED, Helmut Hund Wilovert AFL 30 Series 8 Inverted Trinocular Microscope) with 4×, 10×, and 20× optical magnification, along with different digital magnifications. For the cell confluency dataset, images represented multiple independent experiments, diverse cell lines, and different culture vessels. The full dataset underwent several incremental expansion rounds, where newly captured images were added to the training pool. The final dataset used for the reported model performance consisted of 764 training images, 160 (~20%) validation images, and 81(~10%) fully independent test images. The test set was held out from the start and never used during training, data augmentation, or validation. For the cell count dataset, various cell lines (PC3, MCF7, Raji,) and cell samples (RBCs, PBMCs) were cultured, adherent cells were detached, and cells were loaded into a hemocytometer or KOVA slides at different concentrations, both in the presence and absence of Trypan Blue or Erythrosin B. Cell death was induced by heat shock at 80 °C for 15 min. The dataset also included images of red blood cells, peripheral blood mononuclear cells (PBMCs) isolated as previously described [64], and beads of various sizes (6, 8, 10, and 16 μm) (ThermoFisher; Cat# C16506 and Spherotech Inc; Cat# PPS-6K) (837 images for model training and 93 images for model testing). Images were annotated to generate cell count and live/dead masks for ML. To improve the Count model’s generalization, we applied Gaussian Blur to a representative subset of training images using 20 controlled combinations of kernel sizes (5, 9, 13, 15) and standard deviations (0.0, 0.5, 1.0, 1.5, 2.0). These 244 augmented images were included alongside the original data to simulate out-of-focus conditions commonly encountered in microscopy.

All images were annotated by multiple independent scientists, none of whom participated in model development or training. Annotators used standardized protocol and annotation guidelines, creating a series of independent datasets for training, testing, and validation. All annotations underwent quality review by an independent scientist not involved in either model training or annotation to ensure consistency and accuracy. When discrepancies occurred, a consensus annotation was generated. All testing and validation datasets were independently assembled and randomized by scientists not involved in machine learning model development.

### Cell lines and cultures

MCF7, PC3, HELA, LNCaP, MG63, and HT1080 cells were procured from ATCC (Manassas, VA, USA). IGR-CaP1 cells were provided by Dr. Chauchereau (Gustave Roussy Institute, France) [65]. Cells were maintained in their appropriate media supplemented with 10% FBS in a humidified incubator at 37 °C with 5% CO_2_. All cells were tested for mycoplasma regularly and regularly authenticated by STR profiling.

### IncuCyte® analysis

Cell confluency was measured by IncuCyte® according to the manufacturer’s protocol. Briefly, MCF7 cells, PC3 cells, and IGR-CaP1 cells were seeded in triplicate in 96-well plates. Cells were treated with Docetaxel at different concentrations and were then monitored on the IncuCyte® Live Cell Analysis System (Sartorius, USA). Images were taken at 4× magnification and analyzed by IncuCyte® Live-Cell Analysis Systems.

### SnapCyte™ count

Number and viability of cells were determined by SnapCyteCount and SnapCyte Viability modules, respectively. Briefly, cells were cultured in various cell culture vessels and cell pellets were collected. 10 µL of each sample was individually loaded on a Hausser Scientific™ Bright-Line™ Phase Hemacytometer (Cat.# 026716) or KOVA™ Glasstic™ Slide (Fisher Scientific; Cat.# 22-270141), and the cell numbers were determined by the SnapCyteCount module. For cell viability analyses, equal volumes of Trypan Blue solution, 0.4% (Gibco; Cat.# 15250061) or 0.1% of Erythrosin B (ThermoFisher; Cat.#A14180.14) were added to the resuspended cells in corresponding cell culture media supplemented with 10% FBS before the samples were loaded on a hemocytometer or KOVA™ Glasstic™ Slide (fisher scientific; 22-270141). Cell viability was determined by SnapCyte. For bead analysis, 10 µL of the Cell Sorting Set-up Beads (for UV lasers) (ThermoFisher; Cat.# C16506) or Polystyrene Particle Size Standard Kit, Flow Cytometry Grade (Spherotech Inc; Cat.# PPS-6K) were prepared at different concentrations with distilled water and loaded on a hemocytometer. Bead numbers were determined by the SnapCyteCount module. Four photos per sample were taken by an Apple iPhone 8 and an adapter provided by SnapCyte, mounted on a Nikon ECLIPSE Ts2 inverted microscope at 10× magnification. Region of Interest (ROI) was set up for each photo taken. For comparisons involving multiple users, operators were blinded to sample identity, and the sample processing order was randomized to minimize user-related bias.

### SnapCyte™ confluency

Confluency of cells was measured by SnapCyte Confluency according to the manufacturer’s protocol. Briefly, cells were cultured in various cell culture vessels and cell confluency was determined at different time points by SnapCyte Confluency in photos taken with various phones and SnapCyte universal Smartphone Adapter mounted on Nikon ECLIPSE Ts2 inverted microscope. Region of Interest (ROI) was set up for each photo taken, and results were exported and analyzed. Microscope magnification used and the number of photos taken at different time points in experiments are described in the corresponding figure legends. For comparisons involving multiple users, operators were blinded to sample identity, and the sample processing order was randomized to minimize user-related bias.

### Colorimetric assays

Colorimetric assays (i.e., MTT, WST-1, Cyquant, and Crystal violet assays) were used to determine the viability of MCF7, PC3, and/or IGR-CaP1 at 72 h after treatment with Docetaxel, and subsequently half maximal inhibitory concentration (IC50). Measurements by colorimetric assays were performed according to the manufacturer’s instructions. Briefly, for MTT assay, at 72 h after treatment, the media of the plate were aspirated, after which 50 µL of serum-free media and 50 µL of MTT solution (Sigma-Aldrich; Cat.# M2128) dissolved at 5 mg/mL solution in PBS were added into each well. After incubation at 37 °C for 3 h, 150 µL of MTT solvent (4 mM HCl, 0.1% NP40 in isopropanol) was added to each well. The plate was wrapped in foil and put on an orbital shaker for 15 min at room temperature. The plate was read by a BioTek microplate reader at OD = 610 nm. Docetaxel IC50 in MTT assay was determined by methods described in the “Statistical analysis” section. WST-1, CyQuant assay, and Crystal Violet assay were performed as previously described [64, 66, 67].

### Statistical analysis

The sample size was based on standard practice for in-vitro cell biology experiments. Each condition includes at least 3 biological replicates. Datasets were assessed for normality using the Shapiro–Wilk test and for variance. For experiments with very small sample sizes (*n* too small to reliably test normality) or for datasets that did not pass the Shapiro–Wilk test, we used the Mann–Whitney U two-sided test. Multiple-group comparisons used Kruskal–Wallis. GraphPad Prism software was used to calculate the statistical significance. The threshold of statistical significance was set at *P < 0.05, **P < 0.01, ***P < 0.001, and ****P < 0.0001. All tests were two-sided unless otherwise stated. Error bars represent mean ± SD, and all individual data points are shown where n < 5.

To compare the dose-response curves in Fig.4I, we performed an Extra Sum-of-Squares F-test to evaluate whether IncuCyte, SnapCyte, and MTT could be described by a shared log(IC₅₀) (null hypothesis) or whether allowing each method to have its own log(IC₅₀) significantly improved the model. For all three cell lines, the test did not reject the null hypothesis, indicating that the assays report statistically indistinguishable IC₅₀ values (MCF7: *F*(2,84) = 2.592, *p* = 0.0808; PC3: *F*(2,84) = 0.1605, *p* = 0.8520; IGR-CAP1: *F*(2,84) = 0.4594, *p* = 0.6333).

For Fig.6H, significance was assessed using the Friedman test followed by post-hoc pairwise comparisons: Dunn’s multiple comparison test with correction for multiple testing. All post-hoc p-values were non-significant.

### Determination of IC50

The measured IC50 was calculated using the least square fit of four-parameter sigmoidal curves executed using GraphPad Prism (version 8.4.3) software (GraphPad Software, San Diego, CA, USA, https://www.graphpad.com/scientific-software/prism/). Comparison between the measured and predicted values was performed using the correlation coefficient *R*^2^.

### Doubling time

MCF7 cells, PC3 cells, and HELA cells were seeded in triplicate in 96-well plates at 1.00E + 4 cells/well. Cell confluency was determined by the SnapCyte Confluency model at 24 h, 48 h, 72 h, and 96 h after seeding. Two images were taken per well. Experiments were repeated three times independently, and values are expressed as the mean. Doubling time (Td) of each cell line in each of the three independent experiments was calculated using the equation below, where: Nt is the number of cells at time *t*; N0 is the number of cells initially at time 0; *t* is time (days); gr is the growth rate. Number of Cells at Time *t* (Nt) = N0 * e^(gr * *t*). Growth Rate (gr) gr = ln(Nt / N0) / *t*. Doubling Time (Td) = ln(2) / gr. The doubling times of the three cell lines (PC3, MCF7, and HeLa) were obtained from previously published peer-reviewed studies as well as from documentation provided by cell line distributors. The specific numerical values used in our analysis, along with their corresponding references, are provided in the Supplementary Data (Raw Data Fig. 4L).

### Image analysis platforms

#### SnapCyte™ platform

The SnapCyte™ platform (access via: https://www.app.snapcyte.com/) offers a user-friendly interface accessible via both a web application and a mobile app available to the research community at no cost. Images were captured using a standard microscope equipped with a digital camera or a smartphone adapter - either directly through the mobile app or uploaded via the web interface. Images were then organized into experimental groups and replicates, as predefined by the user on the platform. The appropriate analysis module (cell confluency or cell count) was selected, the region of interest (ROI) was defined, and results were generated within seconds automatically with no user-defined parameters.

For confluency analysis, results were displayed as a percentage, accompanied by an overlay mask to visually confirm segmentation accuracy. For cell counting, the output included cell concentration (cells/mL) and viability percentage. All data, specific time points, or grouped results can be exported as tables containing mean and standard deviation values, or as graphs for easy visualization.

#### Other platforms/models

Segmentation masks were generated using three established, publicly available tools. First, images were processed with the Insilicomab cell confluency (GitHub: insilicomab/cell_confluency) using the authors’ distributed Python workflow. Default parameters were initially evaluated; however, this configuration systematically overestimated confluency, producing near-100% mask coverage across all samples. To correct for this, model parameters were empirically optimized within the software environment by minimizing error relative to manual expert annotations. The final parameter set used for all reported analyses was: mask fill factor of 2, minimum object area of 1500 pixels, block size of 3, and 2 mask refinement iterations. This tuned configuration yielded the lowest error relative to ground-truth manual segmentation and was therefore used for all downstream comparisons. Second, we applied Cellpose (cytoplasm model) within its standard software environment, running all images with the recommended default parameters as described by Stringer et al. [32]. Finally, we used the Confluence Analysis Program (CAP) of Lobsenz and Seidler [53], implemented from the Python script provided in the Supplementary Materials of their preprint, again employing the default configuration outlined by the authors.

Cell counts were obtained using three established, publicly available instance segmentation frameworks. First, images were segmented with Cellpose using both the cyto and cyto3 cytoplasmic models, run in the standard software environment with default settings as described by Stringer et al. [32]. For each image, the number of segmented instances in the resulting label masks was taken as the Cellpose-derived cell count. Second, we applied Omnipose using the reference implementation and default configuration reported by Cutler et al. [56], and again derived cell numbers by enumerating labeled objects in the output masks. Third, we used StarDist in its 2D formulation with the authors’ publicly released implementation and pretrained model suitable for our image modality. Cell counts were obtained analogously by counting individual segmented instances in the StarDist label images.

## Supplementary information

Supplementary Figure Legends

Supplementary Figure 1-01

Supplementary Figure 1-02

Supplementary Figure 2

Supplemental Material-Table 1

Supplemental Material-Table 2

Supplemental Material-Raw data Figs. 2 and 3

Supplemental Material-Raw data Figs. 4–6

Supplemental Material-Raw data Fig 5C

## Data Availability

Raw data related to the present study are included in the manuscript and supplementary materials. The annotated datasets and Machine learning model code are protected by the University of British Columbia's intellectual property and considered trade secrets. Sample data can be available upon reasonable request and can be directed to Dr. Nader Al Nakouzi.
