# Supplementary material for: Enhanced precision in cell culture analytics: leveraging artificial intelligence for unbiased and non-destructive assessment of cell growth and viability
Source: Cell Death Discov. 2026 Apr 13;12:234. doi: 10.1038/s41420-026-03116-9 (PMC13183963; doi:10.1038/s41420-026-03116-9)
Supplement: Supplementary file 1 — Supplementary Figure Legends [file 41420_2026_3116_MOESM1_ESM.docx]

**Supplementary Figure 1. SnapCyte confluency shows a strong correlation with cell counts.** 1.0E+5, 2.0E+5, 4.0E+5, and 8.0E+5 MCF-7 cells, PC3 cells, MG63 cells, and HT1080 cells were seeded in duplicate respectively in 6-well plates. Confluency of cells was determined by SnapCyte at 6 hours after seeding. Results of the three replicates are shown (A, C, E, G). 24 hours later, confluency of cells was determined by SnapCyte, and cells were detached by Trypsin, and cell numbers were determined by Biorad TC20 Automated Cell Counter. Results of the three replicates are shown (B, D, F, H). Five images were taken per well at 4X magnification. Experiments were repeated three times independently and values are expressed in mean.  Comparison between the confluency and cell numbers was performed using the correlation coefficient R2.

**Supplementary Figure 2. Validation of SnapCyte viability on KOVA slides**. Viability of Trypan Blue or Erythrosin-B-stained PC3 or MCF-7 cells loaded on KOVA slides in 64 images were analyzed by SnapCyte and a researcher manually. Results of the model are compared with manual counting. The absolute difference between SnapCyte results and manual counting are plotted and shown.
