# Supplementary figures and images for: Enhanced precision in cell culture analytics: leveraging artificial intelligence for unbiased and non-destructive assessment of cell growth and viability
Source: Cell Death Discov. 2026 Apr 13;12:234. doi: 10.1038/s41420-026-03116-9 (PMC13183963; doi:10.1038/s41420-026-03116-9)

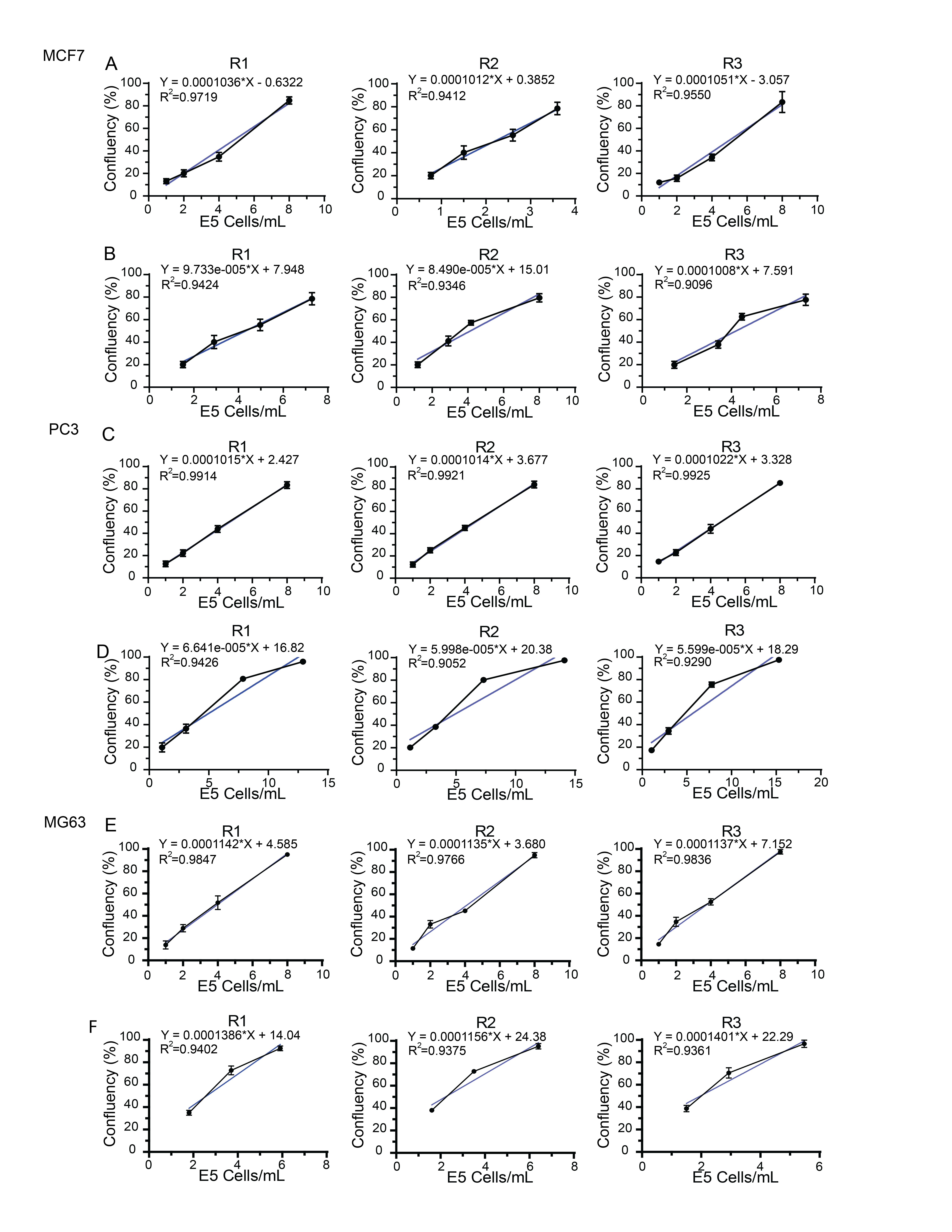

Supplement: Supplementary file 2 — Supplementary Figure 1-01 [file 41420_2026_3116_MOESM2_ESM.png]

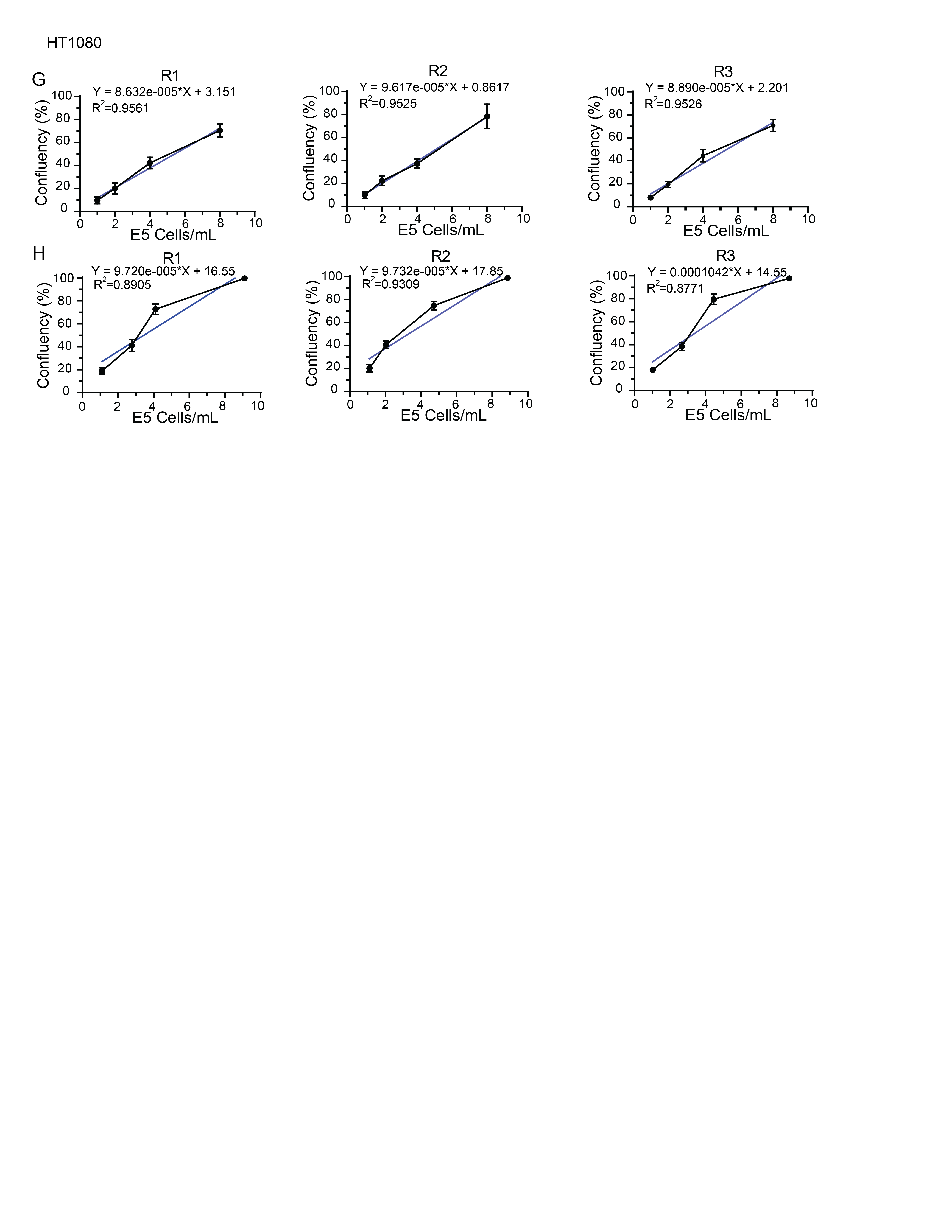

Supplement: Supplementary file 3 — Supplementary Figure 1-02 [file 41420_2026_3116_MOESM3_ESM.png]

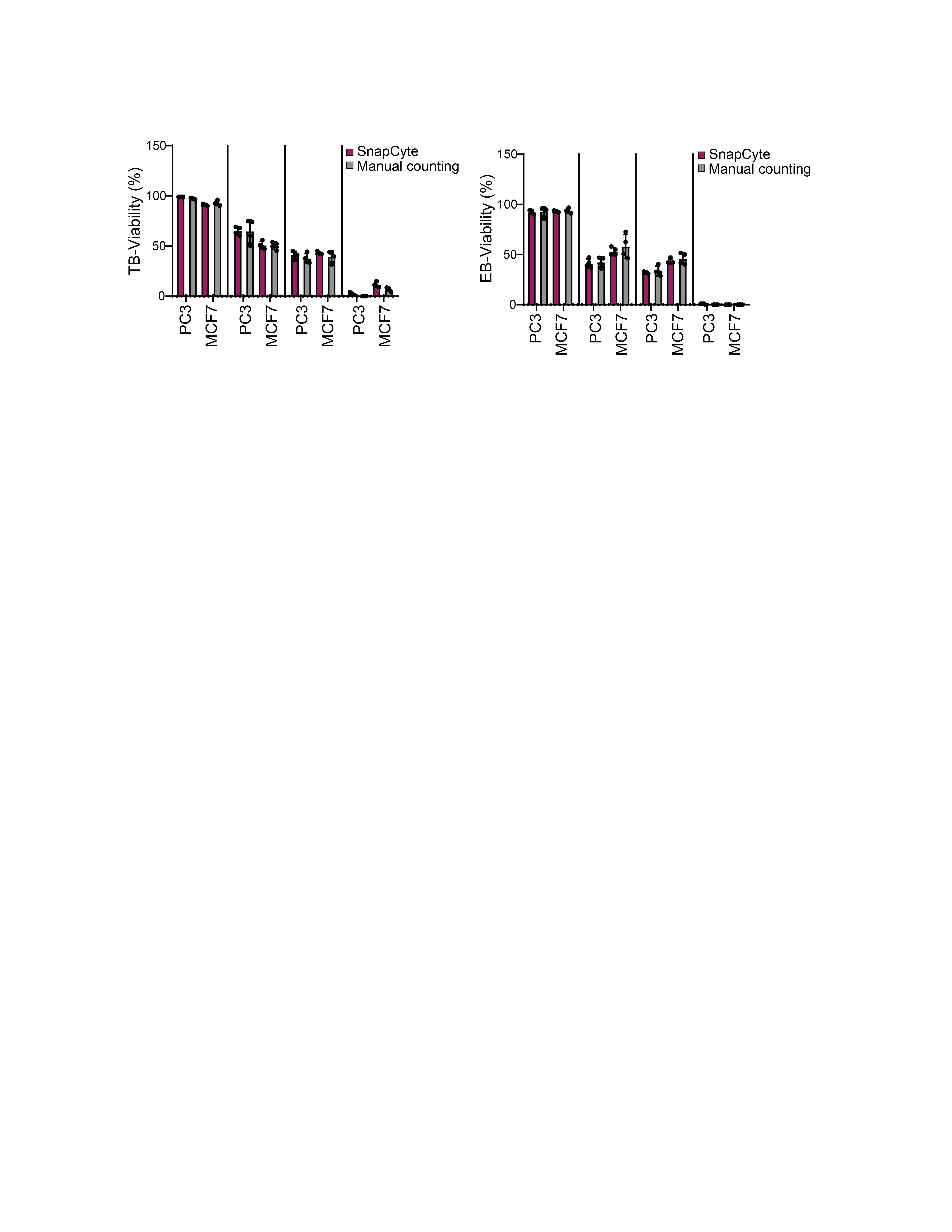

Supplement: Supplementary file 4 — Supplementary Figure 2 [file 41420_2026_3116_MOESM4_ESM.png]
